# Abcès rétropharyngé et la revue de la littérature: à propos de 5 observations

**DOI:** 10.11604/pamj.2020.36.360.24282

**Published:** 2020-08-28

**Authors:** Abdoulaye Keïta, Ibrahima Diallo, Mamady Fofana, Mamadou Aliou Diallo, Mamadou Mouctar Ramata Diallo, Oughaïlou Balde, Alseny Camara, Sory Sacko

**Affiliations:** 1 Service Oto-Rhino-Laryngologie et Chirurgie Cervico-Faciale, Hôpital National Donka, Centre Hospitalier et Universitaire de Conakry, Conakry, Guinée,; 2 Service Oto-Rhino-Laryngologie et Chirurgie Cervico-Faciale, Hôpital Régional de Kankan, Kankan, Guinée,; 3 Unité Oto-Rhino-Laryngologie et Chirurgie Cervico-Faciale, Hôpital Régional de Mamou, Mamou, Guinée

**Keywords:** Abcès rétropharyngé, revue de la littérature, diagnostic, traitement, pronostic, Retropharyngeal abscess, literature review, diagnosis, treatment, prognosis

## Abstract

Nous rapportons 5 cas d´abcès rétropharyngés que nous avons corrélé avec la littérature. Il y avait des enfants ainsi que des adultes. Le corps étranger à type d´arête de poisson a été l´étiologie la plus dominante. La dysphagie accompagnée de douleur, la fièvre et le torticolis ont été les symptômes fréquents. L´abcès rétropharyngé siégeait beaucoup plus en région oropharyngée et hypopharyngée. L´imagerie notamment la tomodensitométrie nous a permis d´avoir des informations précises chez les 3 patients qui en ont bénéficié. L´obstruction des voies aéro-digestives a été retrouvé chez la plupart de nos patients. Seulement 4 patients ont bénéficié d´une incision drainage. Tous les patients ont bénéficié d´un traitement médical. Nous avons enregistré le décès de l´enfant de 2 ans dans un tableau de choc septique à J5 post-opératoire. Nous avons constaté la rareté de cette affection dans notre contexte mais engageant le pronostic vital si le diagnostic et le traitement retardent.

## Abstract (en)

The purpose of this study was to perform correlation of 5 cases and literature review. The study involved both children and adults. The major cause of retropharyngeal abscess was fish bone foreign body. Dysphagia associated with pain, fever and torticollis were the most common symptoms. Retropharyngeal abscess mainly occurred in the oropharyngeal and hypopharyngeal regions. Imaging tests, in particular CT scan, allowed for accurate information in 3 cases. Aerodigestive tract obstruction was found in most of our patients. Only 4 patients underwent incision and drainage. All patients received medical treatment. A 2-year-old died 5 days after surgery due to septic shock. This study highlights the rarity of this condition in our context as well as the importance of early diagnosis and treatment to prevent life-threatening complications.

## Introduction

L´abcès rétropharyngé est une entité infectieuse de l´espace rétropharyngé et un diagnostic rare mais potentiellement mortel [1]. L'abcès rétro pharyngien représente 40 à 80% des infections profondes du cou avec une incidence de 7,5 à 13 cas par an [2]. Cette infection est de diagnostic difficile car la symptomatologie initiale qui regroupe l'irritabilité, la perte d'appétit et la fièvre est non spécifique [3]. Lorsque le diagnostic est posé au début avec une prise en charge adaptée impliquant fréquemment un drainage chirurgical, on a la chance d´obtenir les meilleurs résultats. Par contre, l´évolution se fait par l´aggravation avec l´apparition de dysphagie, d´odynophagie, d´hypersialorrhée ou de détresse respiratoire sévère [1, 2]. D´autres complications plus graves peuvent apparaitre notamment la médiastinite, la péricardite et la septicémie pouvant conduire au décès du patient. Force est de reconnaitre que dans notre contexte sa prise en charge est assez délicate. Le but de ce travail est de corréler ces 5 observations à la revue de la littérature.

## Étude de cas

### Méthodologie

Il s´agissait d´une étude rétrospective (allant du 1^er^ janvier 2010 au 31 décembre 2016) qui nous a permis colligée sur 5 patients, menée au service d´Oto-Rhino-Laryngologie et Chirurgie Cervico-Faciale (ORL-CCF) de l´Hôpital National Donka. Les paramètres suivants ont été étudiés: le profil socio-démographique et les aspects cliniques, paracliniques, thérapeutiques et évolutifs.

### Présentation de cas

#### Observation 1

Il s´agissait d´un garçonnet de deux ans, résidant en milieu rural, sans antécédents particuliers, admis pour détresse respiratoire. Le début de la symptomatologie remonterait environ à 10 jours, marqué par l´ingestion d´un corps étranger au cours du repas, une hypersialorrhée. Des tentatives d´extraction ont été réalisées par les parents qui auraient extrait le corps étranger. Au 5^e^ jour l´odynophagie, la fièvre se sont installés. Au 7^e^ jour, la dyspnée avec tirage se sont installés d´aggravation progressive. C´est ainsi que les parents consultèrent dans une formation sanitaire où l´enfant fut référé dans notre service pour une meilleure prise en charge.

**L´examen à l´entrée notait:** un assez bon état général, un assez bon état clinique, un assez bon état d´hydratation. Dyspnée aux deux temps avec un tirage sus sterno-claviculaire et inter costal. Les téguments et conjonctives bien colorés. Température = 38,5°c; Pouls = 110 b/mn; FR = 32cy/mn; P = 12kg; SO2: 80%.

**A l´examen ORL-CF, on notait:** une légère hyperhémie de l´oropharynx, voussure de la paroi postérieure du pharynx de prédominance hypopharyngé avec une hypersialorrhée. Une hypertrophie des amygdales palatines, non obstructive, cryptique. Une langue saburrale. Hypertrophie des cornets inférieurs bilatérales, traces de rhinorrhée antérieure séro-purulente dans les 2 fosses nasales. Des adénopathies multiples, bilatérales, jugulo-carotidienne, douloureuses, peau en regard saine, inférieur à 3cm, ferme, mobile par rapport aux 2 plans.

**L´examen des autres appareils:** notait une raideur cervicale sans signe d´irritation méningée en faveur d´un torticolis.

**Au terme de l´examen:** un abcès rétropharyngé secondaire à un corps étranger fut suspecté. La radiographie cervicale de face et profil montrait une opacité avec un niveau hydroaérique de siège hypopharyngé (C3-C7). D´où le diagnostic d´abcès rétro-hypo-pharyngé compliqué de dyspnée fut retenu. La biologie a montré une CRP élevée, la VS accélérée et une hyperleucocytose à polynucléaire neutrophile. Notre attitude thérapeutique a été de faire une trachéotomie d´urgence suivie d´une endoscopie. En per-opératoire, nous avons une voussure de la paroi postérieure de l´hypopharynx obstruant le carrefour aérodigestif. Une incision-drainage de l´abcès associée à une aspiration efficace et la pose d´une sonde naso-gastrique d´alimentation. Le corps étranger n´a pas été retrouvé. Le prélèvement du pus fut fait et acheminé au laboratoire pour examen cytobactériologique et antibiogramme.

**En post-opératoire, il a bénéficié du:** ceftriaxone: 100mg/kg par 24 heures répartis en deux prises. Paracétamol perfusable: 20 mg/kg par 24 heures repartis en trois prises. Métronidazole perfusable: 30 mg/kg par 24 heures repartis en deux prises. Bétadine verte en bain de bouche: 3 fois/jour. Les soins de la canule de trachéotomie. A 2 jours post opératoire, le résultat de l´examen cytobactériologique est revenu en faveur des *streptococcus aureus* et celui de l´antibiogramme a noté leur sensibilité aux ceftriaxone, amoxicilline acide clavulanique, ciprofloxacine et gentamicine. Ce qui a permis de poursuivre le schéma thérapeutique. A J5 post opératoire, les suites opératoires ont été marquées par le décès du patient dans un état de choc septique.

#### Observation 2

Patient de 94 ans, cultivateur, résident en milieu urbain, ayant un antécédent d´hypertension artérielle sous traitement et admis pour odynophagie avec fièvre suite à l´ingestion d´un corps étranger (arête de poisson) évoluant depuis deux mois. Le début fut brutal marqué par l´ingestion du corps étranger. Plusieurs tentatives d´extraction par l´envoie des doigts dans le pharynx, réalisées par le patient ont été infructueuses. Une odynophagie s´est installé au 3^e^ jour. Au 5^e^ jour, l´évolution était marquée par la disparition de l´odynophagie suite à une automédication. Deux mois plus tard, réapparait l´odynophagie dans un contexte fébrile. C´est ce qui motiva sa consultation dans le service pour une prise en charge.

**L´examen à l´entrée notait:** un bon était de conscience, coopératif, d´attitude active, fébrile à 38°C, téguments et conjonctives hypocolorés avec un état général peu satisfaisant.

**Figure 1:**
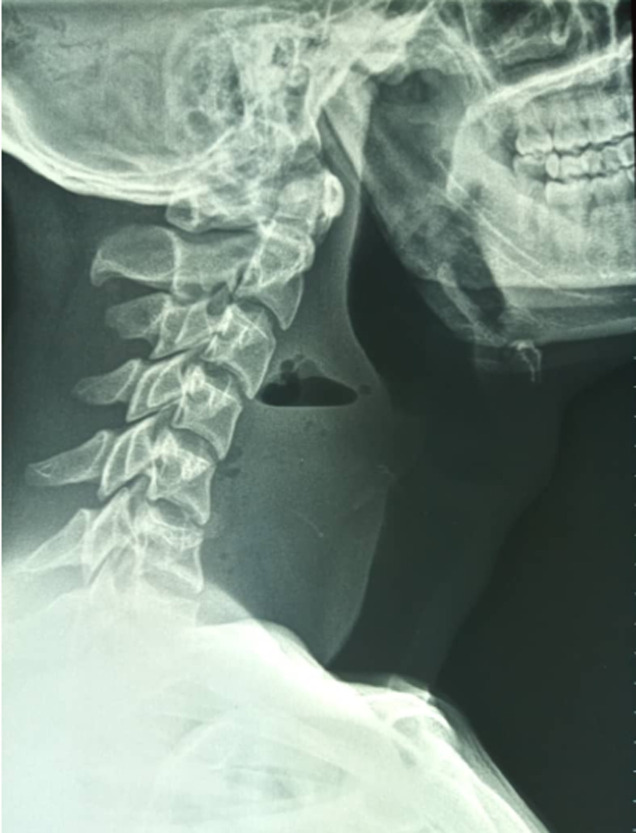
radiographie cervicale profil du 2^e^ patient, montrant une opacité surmontée d´un niveau hydroaérique avec le signe de Miningerode et une opacité linéaire fine, oblique en regard de C4 en faveur du corps étranger

**Figure 2:**
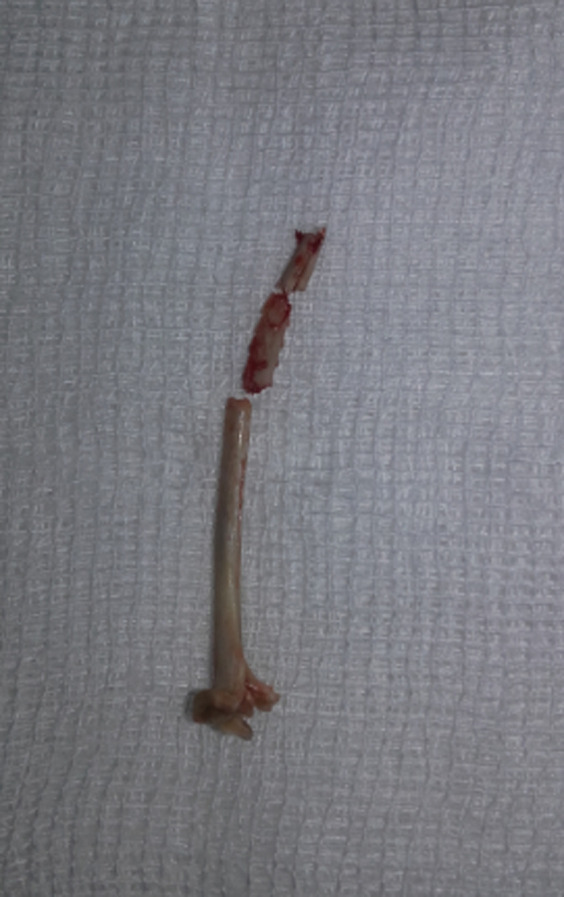
corps étranger extrait chez le 2^e^ patient

**A l´examen ORL-CF, on notait:** une légère stase salivaire, une hyperhémie pharyngée avec voussure de la paroi postérieure du pharynx, une langue saburrale, une hypertrophie des amygdales palatines et des multiples caries dentaires puis édenté par endroits. L´examen des autres appareils notait une raideur cervicale. Au terme de l´examen clinique un abcès rétro-pharyngé secondaire à un corps étranger et amygdalite aiguë furent suspectés. La radiographie cervico-thoracique de profil (Figure 1) a montré une opacité dense avec un niveau hydroaérique (C2-C5) et la présence du corps étranger à type d´arête de poisson. La biologie a montré une CRP élevée et une hyperleucocytose à polynucléaire neutrophile. Le diagnostic d´abcès rétro-oro-hypopharyngé secondaire à un corps étranger (arête de poisson) fut retenu. En per-opératoire l´exploration a noté une voussure de la paroi postérieure l´oropharynx s´étendant à l´hypopharynx, muqueuse en regard plus ou moins jaunâtre. Geste: une incision-drainage associé à une aspiration efficace et pose de sonde nasogastrique d´alimentation. Le corps étranger (Figure 2) a été extrait difficilement. Le prélèvement du pus a été réalisé et la culture était stérile.

**Le patient a bénéficié en post-opératoire:** Ceftriaxone 1g: 2g/24h en intraveineuse pendant 7 jours; paracétamol 1g: 1g/8h en perfusion pendant 7 jours; Dakin cooper: en bain de bouche; Métronidazole 500 mg: 500 mg/8h en perfusion pendant 7 jours. Les suites opératoires secondaires ont été simples et son exeat a été fait à J7 post opératoire avec un traitement par voie orale (Amoxicilline-acide clavulanique 1g, paracétamol 1000mg et Métronidazole 500 mg). A J10 ablation de la sonde nasogastrique. A J15 post opératoire, l´évolution a été marquée par une bonne cicatrisation et la reprise alimentaire par la bouche.

#### Observation 3

Il s´agit d´une patiente de soixante-dix ans, femme au foyer, résident en milieu urbain et avec un antécédent d´hypertension artérielle non contrôlée. Elle a été admise dans notre service pour une dysphagie évoluant depuis une semaine. Le début fut progressif marqué par la survenue d´une vomique associée à une dysphagie dans un contexte fébril. Vu l´aggravation des signes, la famille décida de consulter à l´Hôpital National Donka où le service des urgences médico-chirurgicales l’a orienté dans notre service pour une meilleure prise en charge.

**L´examen à l´entrée notait:** une conscience normale, coopérative, d´attitude peu active avec un état général peu satisfaisant, fébrile à 38°C. Les téguments et conjonctives étaient hypocolorés et les constantes hémodynamiques étaient normales.

**A l´examen ORL-CF, on notait:** une légère voussure de la paroi postérieure de l´oropharynx, un mauvais état bucco-dentaire, une hyperhémie pharyngée, une stase de pus franc, des caries dentaires et édentée par endroits. Une hypertrophie des cornets inferieurs bilatérale et des bouchons de cérumen bilatérale. Des adénopathies multiples II, III et IVa, inflammatoire, ferme, bilatérale, inférieure à 3cm, peau en regard saine, surface lisse, bord régulier.

**L´examen des autres appareils notait:** une diminution des vibrations vocales, des râles crépitants aux 2 champs pulmonaires et une diminution du murmure vésiculaire. A l´issu de l´examen clinique nous avons suspecté une pleurésie et un abcès rétro-pharyngé fistulisé. La TDM cervicale: a montré une collection en rétropharyngé (C3-C6) évoquant un abcès rétro-hypopharyngé fistulisé. La radiographie pulmonaire face a trouvé une pleurésie basale droite de moyenne abondance. Un syndrome inflammatoire biologique a été retrouvé (CRP et NFS). Un avis de la pneumologie fut demandé et qui ont réalisé une ponction évacuatrice. La culture était stérile. Nous avons placé une sonde nasogastrique d´alimentation et l´avions soumis à un traitement médical à base de Ceftriaxone 1g (2g/24h pendant 10j), Paracétamol 1g (1gx3/j pendant 5 jours), Métronidazole 500mg (500mgx3/j pendant 10j) et des solutés en alternance (Sérum salé 0,9%, le Ringer lactate et le glucosé 5%). A 3 Jour de son hospitalisation. L´évolution était favorable. A J4, elle est sortie contre avis médical. C´est ainsi, qu´elle a été perdue de vue.

#### Observation 4

Il s´agissait d´une fille de onze (11) ans, élève, résident en milieu rural, sans antécédent particulier. Admise dans notre service pour odynophagie, notion d´ingestion de corps étranger (arête de poisson), trismus, tuméfaction latéro-cervicale droite, torticolis et dysphonie évoluant depuis six jours associés à la dyspnée. Le début de la symptomatologie a été brutal marquée par l´apparition une d‘odynophagie après l´ingestion accidentel d´une arête de poisson au cours du repas. Les parents l´envoyèrent consulter un tradithérapeute qui a fait des massages cervicaux. L´évolution a été marquée au 4^e^ jour par l´aggravation de la symptomatologie avec aphasie totale et une tuméfaction latéro-cervicale. Ce qui les emmena à consulter dans notre service.

**L´examen à l´entrée notait:** une consciente normale, coopérante, d´attitude active, fébrile à 38,5°C, avec un état général satisfaisant, conjonctives et téguments bien colorés. Les constantes hémodynamiques étaient stables. Poids=35 kg.

**L´examen ORL-CF, on notait:** un trismus à 1.5cm, une voussure oropharyngée s´étendant à la paroi latérale droite et une légère hyperhémie de l´oropharynx. Une tuméfaction latérocervicale droite, inflammatoire, indurée. On notait aussi une limitation de la rotation cervicale. Des adénopathies multiples jugulo-carotidienne droite, inflammatoire, inférieure à 3cm du grand axe, peau en regard saine, ferme, bord libre et mobile.

**Figure 3:**
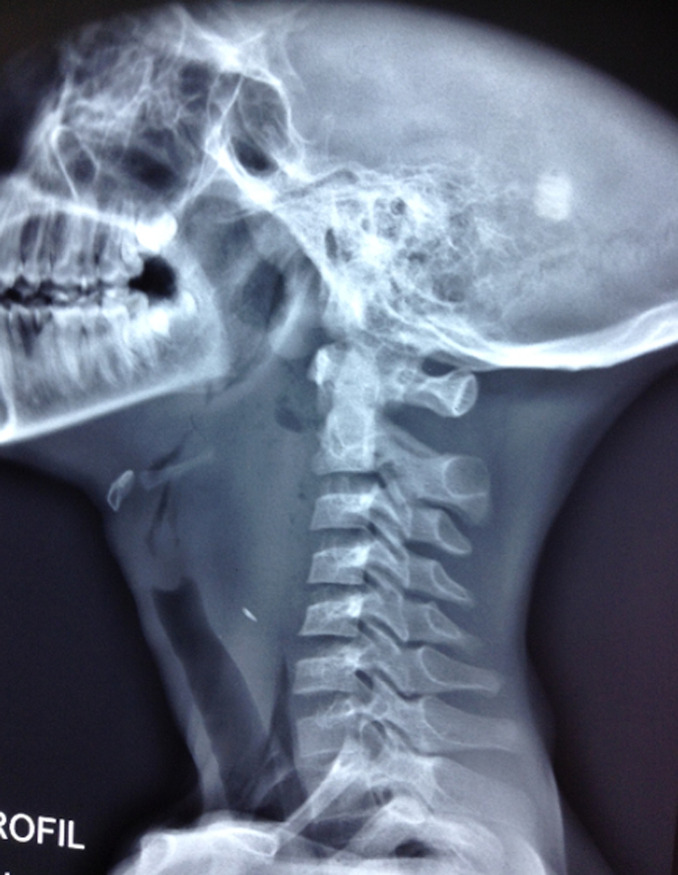
radiographie cervicale profil du 4^e^ patient, montrant une opacité surmontée d´un niveau hydroaérique avec le signe de Miningerode, sans corps étranger visible et obstruant considérablement les voies aériennes supérieures

**Figure 4:**
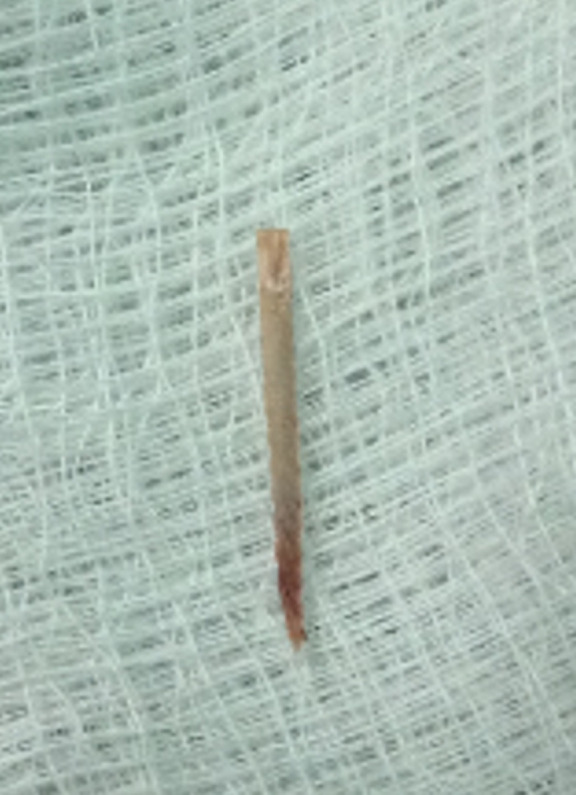
corps étranger extrait chez le 4^e^ patient

**L´examen des autres appareils:** ils étaient normaux et nous avons suspecté un abcès retro-oropharyngé extensive secondaire à un corps étranger. Une radiographie cervicale de profil (Figure 3) a mis en évidence une image radio-opaque arrondie homogène à contours bien nets en regard de C5 (corps étranger) au sein d´une opacité en faveur d´une collection. La biologie a montré une CRP élevée et une hyperleucocytose à polynucléaire neutrophile. En per-opératoire, l´exploration a noté une voussure de la paroi postérieure de l´oropharynx s´étendant à l´hypopharynx et à la paroi latéropharyngé droite. Une incision-drainage associée à une endoscopie et la pose d´une sonde nasogastrique furent réalisées. L´extraction du corps étranger à type d´arête de poisson (Figure 4) a été difficile. Il n´y a pas d´incident ni d´accident per-opératoire. La culture du pus a trouvé le streptocoque Beta-hémolytique du groupe A et des anaérobies. L´antibiogramme était sensible à l´amoxicilline acide clavulanique, azithromycine et rocéphine.

Les soins post-opératoires ont constitué l´administration d´amoxicilline acide clavulanique 1g (1 g/8h pendant 10 jours), du paracétamol perfusable1g (500m g/6h pendant 5 jours), du Métronidazole 500mg (500 mg/8h pendant 10 jours) et du Dakin cooper en bain de bouche. Les suites opératoires ont été marquées par la régression de la symptomatologie avec une persistance de l´odynophagie ayant motivée une seconde endoscopie A J9 post opératoire. En per-opératoire, l´exploration a mis en évidence le bout du corps étranger au niveau de la paroi postérieure de l´oropharynx et l´absence de suppuration. L´extraction de l´arête de poisson a été faite à l´aide d’une pince. Nous avons poursuivi la bi-antibiothérapie et le bain de bouche. Les suites opératoires secondaires ont été simples. Son exeat a été fait à J3 post opératoire de la 2^e^ endoscopie avec une ordonnance par voie orale à base de l´amoxicilline-acide clavulanique, de paracétamol et de collutoire. Le suivi a été marqué par une bonne amélioration clinique au bout d´un mois.

#### Observation 5

Il s´agissait d´un enfant de trois ans, élève, résidant en milieu rural et sans antécédent particulier. Le début de la symptomatologie remonterait d´environ 1 semaine par l´ingestion d´un corps étranger (arête de poisson) au cours du repas. Des multiples tentatives d´extraction furent réalisées et ont été sans succès. Puis s´ajoute l´odynophagie, la fièvre et l´hypersialorrhée au bout de 3 jours. Les parents l´envoyèrent consulter dans une formation sanitaire où un traitement non documenté a été instauré. Vu l´aggravation de la symptomatologie par l´asthénie physique à partir du 5^e^ jour, les parents consultèrent de nouveau dans la même formation sanitaire où il fut référé à l´Hôpital National Donka. Le service des urgences médico-chirurgicales demanda un avis ORL. Ainsi, il a été décidé de la transféré en ORL pour meilleure prise en charge.

**L´examen à l´entrée a noté:** un bon état de conscience, une asthénie physique bavant sur ses vêtements, fébrile à 38,5°C, les conjonctives et téguments étaient bien colorés avec un état général peu satisfaisant (P = 15kg).

**A l´examen ORL-CF, on notait:** une langue chargée, une hyperhémie de l´oropharynx, voussure de la paroi postérieure du pharynx et une hypersialorrhée. Une hypertrophie des cornets inférieurs. Des micro-adénopathies multiples, II et III droite, inflammatoires, mobiles, peau en regard saine, fermes. Une tuméfaction latéro-cervicale droite haute, sensible, lisse, mobile, ferme, non fluctuante et mesurant environ 4cm de grand diamètre.

**L´examen des autres appareils a noté:** ils étaient sans particularités. Au terme de l´examen clinique nous avons pensé à un abcès rétropharyngé extensive à la paroi latéropharyngé secondaire à un corps étranger. La biologie a montré un syndrome inflammatoire. La radiographie cervicale de profil a montré une image radio-opaque arrondie homogène à contours bien nets en regard de C5. La TDM cervico-thoracique a noté une collection liquidienne en rétropharyngé s´étendant à l´espace latéropharyngé droite, une infiltration des tissus mous et un médiastin normal. En per-endoscopie, nous avons objectivé une voussure de la paroi postérieure de l´oropharynx d´étendant à la paroi latéropharyngé droite et discrètement à l´hypopharynx. L´incision-drainage de l´abcès fut réalisée avec extraction de l´arête de poisson et la pose d´une sonde nasogastrique. Le pus prélevé d´aspect chocolaté a été acheminé pour examen cytobactériologique qui a trouvé des diplocoques et des bacilles à Gram positif (+). L´antibiogramme a noté leur sensibilité à l´Ofloxacine et à la Rocéphine.

Les soins post-opératoires étaient composés du Ceftriaxone 1g (100 mg/kg par jours pendant 5 jours), Paracétamol 500mg (60 mg/kg toutes les quatre heures pendant 5 jours) et Prednisolone 4mg (1 ampoule par jour pendant 5 jours, instaurée après 3 jours d´antibiothérapie) et un Collutoire (2 pulvérisations x 3/j) L´antibiothérapie initial n´a pas été modifié après antibiogramme. Les suites opératoires ont été simples. Le patient a été sorti de l´hôpital à J6 post-opératoire avec une ordonnance médicale par voie orale à base d´amoxicilline-acide clavulanique, de paracétamol 200mg. Le patient a été revu au contrôle après une semaine puis à un mois. Il était guéri de son affection.

## Discussion

Les abcès rétropharyngiens sont une entité bien connue chez l´enfant. Mais ils sont rares chez l´adulte bien que peuvent survenir et présenter des complications graves [4]. La fréquence des abcès rétropharyngés dans notre série était de 60% chez les enfants et de 40% chez les adultes. Ceci s´expliquerait par l´exposition au corps étranger et les infections fréquentes de la sphère ORL chez les enfants. Ce constat a été fait par de nombreux auteurs [2, 5]. L'espace rétropharyngien peut être infecté de trois façons. La *première*, l'infection se propage par contiguïté d´une infection de la muqueuse pharyngée affectant les ganglions rétropharyngiens (ganglions de Gilette). La *deuxième*, l'espace est inoculé directement par un corps étranger pénétrant. La *troisième*, l´infection peut être d´origine lymphatique par drainage de la lymphe des zones infectées du pharynx ou de l´oreille moyenne dans les ganglions de l´espace rétropharyngien [6]. Bien que les nœuds lymphatiques rétropharyngés n´existent plus à l´âge adulte, l´infection peut survenir et toucher d´emblée les fascias alaire et prévertébral [5]. Il existe également des causes iatrogènes, notamment l'instrumentation avec laryngoscopie, l'intubation endotrachéale, la chirurgie et l'endoscopie [1]. Il ne faut pas occulter les lésions engendrées sur le pharynx lors des tentatives d´extraction des corps.

Dans notre série, la deuxième et troisième étiopathogénie seraient responsables des abcès rétropharyngés. Chez quatre sur cinq de nos patients, la survenue de l´abcès rétropharyngien était due aux corps étrangers à type d´arête de poisson. De nombreux confrères ont fait le même constat [2, 5]. Seule la troisième observation avait une étiologie mal élucidée et la 3^e^ étiopathogénie expliquerait la survenue d´abcès rétropharyngé. Par contre dans l´étude menée par Fédérici S *et al.*[7], qui a colligé sur trente et un patients, tous les cas d´abcès rétropharyngés étaient secondaires à une infection des voies aérodigestives supérieures (angine, rhinopharyngite, otite moyenne aigue et une mastoïdite). A l´issu de toutes ces remarques, nous avons constaté que l´étiologie des abcès rétropharyngés sont multiples et que dans notre série l´étiologie différait d´un cas à un autre mais majoritairement représentée par les corps étrangers à type d´arête de poisson.

Le diagnostic clinique de l´abcès rétropharyngé peut être difficile. La symptomatologie clinique est variable et non spécifique incluant une dysphagie, une odynophagie, une fièvre, une dyspnée, une douleur cervicale, un abcès cervical, un trismus [1, 5]. Ces signes ont été rapportés par Thomas *et al.*[8]. Dans la présente étude, la dysphagie accompagnée de douleur le plus souvent suivies de la fièvre ont été les symptômes les plus rapportés avec respectivement 100 et 60 % et le torticolis était plus évident à l´examen clinique. Cette symptomatologie qui est mal vécue par le patient, l´oblige à consulter dans une formation sanitaire. Par contre Fédérici S *et al*. [7] ont cité le torticolis et la fièvre (100%) associée à l´odynophagie (32,3%) comme les symptômes les plus dominants dans leur série. Le torticolis est un signe qui s´installe généralement à un stade avancé de l´abcès rétropharyngé [9]. Parmi nos patients, un enfant de deux ans a été reçu avec une dyspnée intense et a nécessité la réalisation d´une trachéotomie d´urgence. La survenue de cette dyspnée est secondaire à l´obstruction de la filière aérienne et est considérée comme une des complications létales selon Doumbia *et al*. [5] et de nombreux autres auteurs [6, 7].

L´examen physique permet d´orienter le diagnostic en révélant un bombement de la paroi pharyngé postérieure [1, 5]. Dans notre série, la voussure de la paroi postérieure a été retrouvée chez tous les patients. Le siège oropharyngé était dominant par rapport à celui hypopharyngé et aucun cas d´abcès rétro-nasopharyngé n´a été trouvé. Cette voussure a été aisément confirmée en per-endoscopie. Dans l´étude de Doumbia *et al*. les symptômes évocateurs ont été la fièvre, la dysphagie et l´abcès cervical [5].

Les radiographies du cou de profil sont généralement l´examen d'imagerie de choix dans l'évaluation initiale d'un abcès rétropharyngé présumé, en particulier chez les jeunes enfants. Elles ont l'avantage d'une exposition aux rayonnements plus faible et ont tendance à être mieux tolérées par les patients qui présentent des signes de compromis des voies respiratoires. Par contre la TDM du cou avec contraste intraveineux est la meilleure étude d'imagerie pour évaluer les patients atteints d'un abcès rétropharyngé [1] mais qui sont impossibles à réaliser dans le contexte de l´urgence [6]. De nos jours, l´IRM occupe une place importante dans le diagnostic des abcès rétropharyngé [6] mais il reste un examen très couteux et moins accessible dans notre contexte. La sensibilité du scanner pour détecter un abcès rétropharyngé varie dans la littérature de 64% à 100% [1]. Par contre pour la radiographie cervicale, elle est de 88% [10]. Dans notre étude, tous nos patients n´ont pas pu bénéficier de la TDM vue l´état d´urgence et le bas niveau socio-économique dans notre contexte. Deux patients ont fait la radiographie cervicale et trois la TDM avec injection de produit de contraste. Ces résultats étaient en faveur d´une collection dans la région rétropharyngée extensive aux parois latérales du pharynx parfois avec le signe de Miningérode. La fiabilité de ces examens complémentaires notamment la TDM a été décrits aussi par de nombreux auteurs [1, 2, 5].

Un syndrome infectieux biologique sévère est souvent associé, mais il n´existe pas de paramètre biologique pouvant renseigner sur le stade de l´infection [6]. Pour tous nos patients le syndrome inflammatoire était présent notamment la CRP élevée et une hyperleucocytose à polynucléaire neutrophile. Certains confrères ont fait le même constat [5, 7]. Les germes retrouvés dans la littérature sont des aérobies (*Staphylococcus aureus*, Streptocoque Beta hémolytique, Streptocoque pyogène, *Haemophilus influenzae*), des anaérobies (Bacteroïdes et Veillonella). Dans cette série, le Streptocoque Beta-hémolytique du groupe A, le *Staphylococcus aureus*, les diplocoques à Gram positif et les anaérobies étaient les germes isolés. Quasiment, les mêmes germes ont été retrouvés par certains auteurs [5, 10]. L´antibiogramme a cité des molécules (Ceftriaxone, Cefotaxime, Azithromycine et l´Amoxicilline acide clavulanique) déjà entamées en première intention ce qui a permis leur continuation. Il faut signaler que le Métronidazole accompagnait l´antibiothérapie. Sharma *et al*. [8] ont procédé à la même démarche pour le choix de l´antibiothérapie et ont rapporté quasiment les mêmes molécules. Nous avons préféré le Ceftriaxone et l´Amoxicilline acide clavulanique à cause de leur biodisponibilité. Le bain de bouche a été prescrit pour des soins buccopharyngés et permettait d´aseptiser le foyer infectieux.

Le traitement est médicochirurgical et la voie d´abord dépend de la localisation et de l´extension de l´abcès. La voie transorale est utilisée par la majorité des équipes [1, 5]. De même que dans la présente étude. L´anesthésie générale nous a permis confort et sécurité au cours de l´incision-drainage. Les corps étrangers notamment à type d´arête de poisson ont été difficilement extraits chez le deuxième, quatrième et cinquième patient. Tandis que chez le premier patient le corps étranger n´a été retrouvé ni à l´imagerie ni en per-opératoire. Il faut reconnaitre que l´extraction de ces agents étiologiques, nous a réconfortées dans notre attitude thérapeutique. Nous avons procédé aussi à la mise en place d´une sonde nasogastrique d´alimentation chez tous les patients et elle permettait en même temps de protéger les voies ariennes. La même attitude a été adoptée par de certains auteurs notamment par Doumbia *et al*. [5]. Seul le premier patient a bénéficié d´une trachéotomie à cause de sa dyspnée sévère. Pour le traitement médical, il n´existe pas de divergence avec les principes dictés dans la littérature [1, 6].

L'abcès rétropharyngien entraîne un taux élevé de morbi-mortalité, en raison de son association avec l'obstruction des voies respiratoires, la pneumonie, la médiastinite, la thrombose veineuse jugulaire, la fasciite nécrosante, la septicémie et l'érosion de l'artère carotide. Nous avons noté des suites opératoires secondaires marquées par le décès du premier patient dans un tableau de choc septique. Pareil pour l´équipe de Coulthard M *et al*. [10] qui a trouvé 2 cas de décès sur 31 patients et par contre, Doumbia *et al*. n´ont pas noté de cas de mortalité [5]. Un diagnostic et une prise charge précoce bien menés pourrait réduire ce taux de mortalité [1].

## Conclusion

L´abcès rétropharyngé est urgence médico-chirurgicale engageant le pronostic vital. Il survient à tout âge mais plus fréquente chez l´enfant. Les corps étrangers à type d´arête de poisson constituent l´étiologie traumatique la plus fréquente notamment chez l´adulte. Nos patients ont développé une symptomatologie plus ou moins spécifique de l´abcès rétropharyngé. L´imagerie a été d´intérêt capital dans le diagnostic. Ses complications sont graves et sa prise en charge doit être précoce et adéquate pour réduire le taux de mortalité.
